# Optimization of Computer Web Page Interface Based on BP Neural Network Algorithm and Multimedia

**DOI:** 10.1155/2022/6213718

**Published:** 2022-05-25

**Authors:** Yan Ma

**Affiliations:** Information Technology Center, Wuxi Vocational Institute of Arts & Technology, Wuxi 214200, China

## Abstract

In web design, we need to make full use of computer multimedia technology, which can effectively improve the quality of web design and provide greater convenience. The neural network needs to calculate a large amount of training data in image optimization, and the calculation speed cannot keep up with real-time data processing, resulting in the problem of poor quality of optimized images. This paper analyzes the problems existing in the traditional optimized BP neural network algorithm and puts forward an optimized BP neural network image optimization method which combines the increase of momentum term with the adaptive adjustment of learning rate. This method can speed up the iteration speed and jump out of the situation of premature local minimum. The test results show that the user satisfaction of the text visual effect of the web interface optimized by this method is more than 98% and the user satisfaction of other methods is only about 90%. The visual satisfaction of the web interface optimized by this method is significantly higher than that of the comparative method. The web interface visual optimization effect of the method in this article is better, and it can meet the satisfaction requirements of most users.

## 1. Introduction

**Figure 1:**
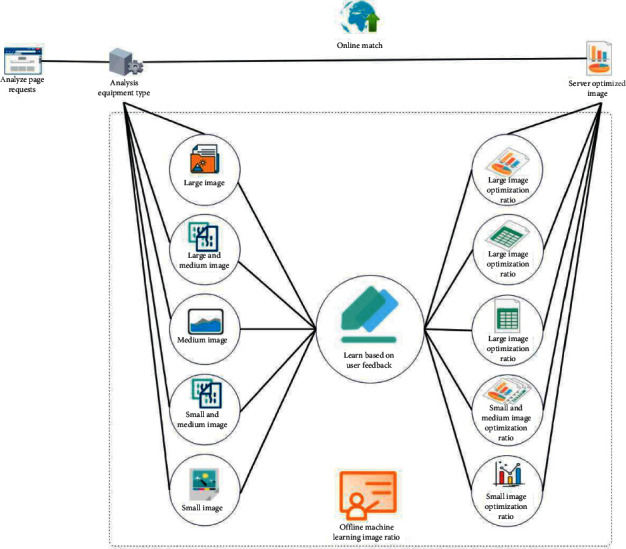
Optimization process.

In the age of the Internet, the amount of information in network data is increasing and information technology is widely used in all walks of life with unique advantages. In terms of network design, computer multimedia technology can effectively improve the gold content and overall beauty of web content for practical use in the latest technology, provide comfort to web designers, reduce workload, and improve the quality of web design [1]. As shown in Figure 1, the application of computer multimedia technology in web page design is an inevitable choice, but in practical applications, there are still many issues to be further realized and improved. From this point of view, it is very important to strengthen the application research of computer multimedia technology in web design and it is helpful to provide reference basis for the follow-up work [2]. The main idea of the BP (error backpropagation) algorithm is to first select a set of weights arbitrarily. The learning process of this group of weights is divided into two stages: the forward propagation of the signal and the backward propagation of the error. In the forward propagation phase of the signal, the input variables are transferred to the output layer through the input samples of the input layer and the hidden layers, for obtaining the information we want in the output layer. During this calculation, the weights and sample offsets of each network sample must be consistent and the neuron states of layers are transmitted through related activation functions [3]. If the sample calculation error cannot be converged, then the error backpropagation stage will be carried out. In the backpropagation phase of the error, contrary to the forward propagation process of the signal, the output end is regarded as the beginning and the error direction is propagated forward layer by layer. By transmitting the output error back, the error is distributed to all units in the original layer, so as to further obtain the error signal of each layer unit and then correct the weight of each unit. Through repeated training, the weight of network samples and the offset value of samples are continuously modified until the predetermined error accuracy is reached [4]. From this point of view, it is very important to strengthen the application research of computer multimedia technology in web design, which is helpful to provide reference for the follow-up work.

## 2. Literature Review

Guo et al. proposed that page rendering can be achieved by using CDN (Content Delivery Network) and can reduce the download time of each file in the web page [5]. Yang proposed a web browser, which can quickly display web pages containing dynamic objects even if the rendering process is completed in the proxy server. The proxy server presents the dynamic and static parts, respectively, and sends the rendering results of the dynamic part and the optimized rendering results of the static part to the web browser [6]. A web application framework was proposed by Jiang and Wang to generate rendering results; for example, the DOM tree in the web server sends the results to the web browser, and after sending the presentation result, it sends the Ajax event to the web browser [7]. Chen and others discussed how to divide the roles between the server and the browser, for effective processing and presentation results [8]. In academia, Yiyue and others proposed the SACC algorithm based on the queue scheduling model. The SACC algorithm can adjust the order of the request according to the priority of the change request. The SACC algorithm can reduce the request waiting time and optimize the front-end performance to a certain extent. The adaptive method of task scheduling strategy has become an important research field, especially in the optimization of distributed systems [9]. Liu and Zhang proposed the Markov request queue model which considers resource sharing between VMs and several types of failures [10]. For the queue buffer size and uncertainty value function, Deng et al. proposed that through job scheduling, the adaptive action selection method of reinforcement learning algorithm and queuing theory can be realized [11]. At present, the bottleneck between high-speed CPU and low-I/O hardware is still very serious, and most of the proposed methods are used to improve the throughput of high concurrency and reduce network delays to improve I/O performance. A method to improve I/O performance through a distributed file system is also proposed. Like disk I/O scheduling, in a distributed file system, continuous I/O requests are recalculated and reorganized through some algorithms and then submitted to the local file. Due to the increasing popularity of machine learning, it can be combined with performance optimization. Regarding machine learning, in academic terms, Lu and others proposed a distributed learning algorithm and used reinforcement learning as the framework of their work. In their shared ordinal learning method, each scheduler has a utility table [12]. Hajipour and others proposed the use of adaptive reinforcement learning algorithms, to improve task execution with lower computational complexity. This method uses the combination of RL and neural network to support scheduling [13]. Feng et al., based on the 0-1 scoring model, proposed the Difficulty Matching Method (Optimal Item Difficulty, OID). This is the original form of computer adaptive testing. However, this method relies on the choice of initial test items, and the test efficiency is low. Then, based on the relationship between the ability level of subjects and the amount of project information, Lord proposed the maximum Fisher information (MFI). The hypothesis of the maximum information method is that the greater the amount of information in the project, the closer the evaluation results of the subjects are to the real results [14].

As far as the current research is concerned, neural network needs to calculate a large number of training data in the process of image optimization. When the computing speed cannot keep up with the real-time processing speed of data, it will lead to the problem of poor image quality. This paper analyzes the problems existing in the traditional BP neural network optimization algorithm, optimizes the traditional BP neural network optimization algorithm, and combines it with adaptive adjustment learning to effectively accelerate the iteration speed. This method can give full play to the effect of BP neural network algorithm to a greater extent, accelerate the iteration speed, and also jump out of the situation of premature local minimum, so as to greatly improve the calculation speed and obtain high-quality computer web page interface optimization.

## 3. Analysis of Computer Multimedia Technology in Web Design

### 3.1. Web Page Video Processing Technology

The use of computer multimedia technology in web page design can help in obtaining videos from digital cameras and CDs. Due to its own characteristics, the amount of internal data is relatively large. Based on the current digital TV image NTSC and SIF standards, it can be found that the amount of data per frame in the digital TV image is as high as 2038 KB and the data traffic per second is around 62.7 MB. Uncompressed videos are very difficult to transmit on the network, and it is easily affected by network resources, seriously affecting the normal use of the network. Therefore, in web page design, it is necessary to fully consider the choice of video processing technology [15].

### 3.2. Digital Audio Processing Technology

There are many audio files on the Internet, and the formats of these audio files are mostly MP3, WAV, WMA, and AAC. Digital technology is one of the more advanced technologies in multimedia technology, and it has been applied to web design much earlier. In web design, there are various ways to obtain digital audio resources, which can be downloaded or purchased online or obtained from the audio material library. Based on this, in web design, it needs to be clarified that sound is a carrier of information dissemination, which integrates the audio and video organically and can effectively improve the effect of information presentation [16].

### 3.3. Web Graphics and Image Processing Technology

Graphics and images are equally indispensable elements in web design. The multimedia information contained in them is relatively rich, and through image compression and processing, two-dimensional and three-dimensional images can be generated. Image acquisition is mostly achieved through scanning technology and digital acquisition technology, and there are various image processing methods, such as the more common Photoshop software. It can be adjusted in all directions according to the requirements of the image, including image exposure, contrast, softening, and cropping, also can trim the image, and select the appropriate file format to input. The more common image formats include jpg, GIF, amp, JPEG, etc. In web design, through colorful images, it can make web content more vivid and give people a visual impact [17].

### 3.4. Optimization Method of the BP Neural Network

**Figure 2:**
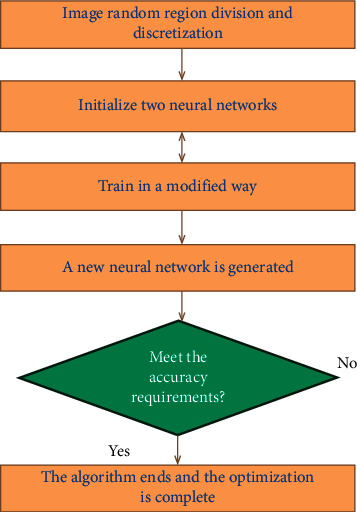
Image optimization process based on the optimized BP neural network.

The overall idea of optimizing the image optimization of BP neural network is as follows: First, we establish two three-layer BP neural network models, where the number of nodes in the first two layers is 4 and the number of nodes in the third layer is 1. We select the initial value of the connection weight between the layers, take the value of each neural unit in the BP neural network as the regional value, perform iterative operations, and obtain the output result as the optimal decision result. Finally, we perform equivalence class aggregation according to the optimal decision result to complete the image optimization. The algorithm flow chart is shown in Figure 2.

#### 3.4.1. Traditional Optimization Methods

Modifying the weight learning formula of BP neural network is the main method to improve the traditional neural network, and the main improvement methods are as follows:

(i) Increasing the momentum term: when the traditional BP neural network algorithm adjusts the weight and offset value, regardless of the direction of the gradient before the error at a certain moment, we only consider the gradient descent direction of the error at a certain moment and make adjustments, so that it is easy to cause oscillations during training, it is easy to increase the number of iterations, and it is easier to fall into a local minimum [18]. Therefore, in order to speed up the calculation of the neural network, some scholars have proposed adding a momentum term to adjust the weight. The general expression of the weight adjustment expression can be expressed as
  (1) $$\begin{array}{c} \Delta W\left(t\right)=\eta \frac{\partial E}{\partial W\left(t-1\right)}+\alpha \Delta W\left(t-1\right). \end{array}$$
Here, *W* represents the weight matrix of a certain layer and *α* is the momentum coefficient. Expression (1) can also be further optimized, which can be optimized as
  (2) $$\begin{array}{c} \Delta W\left(t\right)=\eta \left(1-\alpha \right)\frac{\partial E}{\partial W\left(t-1\right)}+\alpha \Delta W\left(t-1\right). \end{array}$$
(ii) Learning rate adaptive adjustment algorithm: in the algorithm of neural network, when the important parameter learning rate *η* is small, although it can prevent the training from falling into a local minimum, stable convergence of training can be guaranteed, but the speed of convergence becomes slower. Conversely, when the learning rate *η* becomes larger, the training convergence speed is greatly improved, but it is easy to repeat the training and fall into a local minimum. Therefore, in the neural network algorithm, the balance of the learning rate *η* must be considered. But the learning rate in the ordinary BP neural network algorithm is a fixed value, and many scholars have shown that the fixed learning rate causes more problems in practical applications. On a flat error surface, in order to speed up the training iteration speed, the learning rate *η* value is increased. When there are more changes in the error surface, the learning rate *η* is reduced to prevent it from falling into the local minimum. Therefore, how to adjust the learning rate adaptively in the algorithm has become a hot spot of improved algorithm research [19].
(iii) Combining the increase of momentum term with adaptive adjustment of the learning rate: that is, the learning rate *η* and momentum coefficient *α* in the formula (1), At the same time, in order to ensure the stable convergence of training, self-regulation according to the actual situation of convergence can greatly improve the training speed. To solve this problem, some scholars have also put forward some reasonable methods [20].
(iv) Other improvement methods: according to the second differential term of the error, the BP neural network is modified to a certain extent by modifying the selection method and topology of the activation function on each neuron, and a great improvement is made in one aspect.

#### 3.4.2. Improved Optimization Methods

This algorithm uses a combination of increasing momentum terms and adaptive adjustment of the learning rate. The optimization algorithm of BP is given. This optimization algorithm, compared with the optimization algorithm introduced earlier, reduces the amount of changing parameters and extracts *η* and *β* as one parameter, so there is

(3) $$\begin{array}{c} \Delta W\left(t\right)=\beta \left(\eta \frac{\partial E}{\partial W\left(t-1\right)}+\alpha \Delta W\left(t-1\right)\right). \end{array}$$

At a certain moment, the value of *t* and *β* is adjusted according to the difference Δ*E*(*t*) between the error of this iteration and the error of the previous iteration. The specific method is as follows: if Δ*E* ≥ 0 which indicates that the error has further increased after this iteration, it is defined as invalid and the value of *β*_remains_ unchanged [21]. If Δ*E* < 0, it indicates that the error has been reduced after this iteration and hence defined as valid; the *β* value is modified to

(4) $$\begin{array}{c} \beta \left(t\right)=\beta \left(t-1\right)\left(1+\lambda \right). \end{array}$$

Here, *λ* is any positive number between 0 and 1.

However, this modification does not greatly improve and optimize the training speed, and the only reduction is the amount of calculation and parameters. Therefore, in order to optimize the algorithm, the ideas of evolution theory must be added to this algorithm. Use purposeful incremental fine-tuning to adjust the node weight, judge and select the fine-tuning weight, and determine which group to keep. The algorithm extracts two network samples for training at the same time. The new value of a parameter in the algorithm is weighted by Gaussian noise and used as the weighted value of the new network. When the training of each layer is completed, make judgment and selection, select a better network weight, weight the network weight with Gaussian noise for the second time, and repeat the training until certain requirements are met [22].

### 3.5. Big Data Processing Based on Computer Interaction Technology

The method of big data information fusion and data scheduling is used to complete the information integration processing during interface interaction, and the time-domain function of big data distribution during interface interaction is established as follows:

(5) $$\begin{array}{c} {\dot{w}}_{j}^{i}=l_{j}^{i}\left(w_{j}^{i},v_{i},v_{j}\right), \end{array}$$

where *j* and I are dimensions of sampling nodes and the underlying database of interface interaction, respectively. *l*_*j*_^*i*^ is the data node interactively transmitted at the JTH sampling node; *w*_*j*_^*i*^, V, and *L* are the output clustering vector, large data volume, and feature classification, respectively. ${\dot{w}}_{j}^{i}$ is consistent with the feature distribution of interface interaction level *v*_*i*_ ∈ *Q*^*n*×*N*^. In order to obtain the characteristic distribution of the output clustering attributes of big data clustering, cross-compilation was used to calculate the clustering information according to the following method:

(6) $$\begin{array}{c} w={\left[\ldots w_{i}\ldots w_{j}^{i}\ldots \right]}^{z}\in Q^{n\times N}, \end{array}$$

where *n*, z, and *Q* are the order, fixed constant, and interface interaction parameters set by interface interaction, respectively. In order to obtain the clustering central function of big data, the interclass dispersion of big data distribution is used to calculate the method of feature classification and recognition:

(7) $$\begin{array}{c} \dot{w}=l\left(w,v\right). \end{array}$$

To obtain the statistical independent variable of the underlying big data in interface interaction, it needs to be calculated according to the statistical feature analysis method:

(8) $$\begin{array}{c} v=\left(v_{i},v_{j},...,v_{N}\right)\in Q^{m\times N}. \end{array}$$

Through the method of big data fusion output, the phase trajectory distance between the center vectors *b*_*i*_ and *b*_*j*_ of interclass clustering of the data information flow of the underlying interface is calculated:

(9) $$\begin{array}{c} B\left(b_{i},b_{j}\right)=\frac{b_{i}\cdot b_{j}}{\left\|b_{i}\right\|\cdot \left\|b_{j}\right\|},\\ W=\left\{w_{1},w_{2},...,w_{n}\right\}\subset Q^{t}. \end{array}$$

In order to obtain distributed statistical results of clustering of large data information in interface interaction, the autocorrelation semantic feature grouping method is used to calculate

(10) $$\begin{array}{c} A=\left\{V_{ij}|i=1,2,...,e;j=1,2,...,t\right\}, \end{array}$$

where *V*_*i*_ is the large data volume of the JTH clustering center and *E* and *T*, respectively, represent the total dimension of the underlying database of interface interaction and the total number of sampling nodes. In order to obtain the scheduling function *P*_*m*_(*V*, *A*), the real-time interface interactive data of big data without difference features is scheduled as follows:

(11) $$\begin{array}{c} P_{m}\left(V,A\right)=\sum_{f=1}^{n}\sum_{i=1}^{e}\mu_{if}^{m}{\left(b_{if}\right)}^{2}, \end{array}$$

where *m* and *μ*_*if*_ are the spatial distribution dimension of data clustering and the scheduled data, respectively, and (*b*_*if*_)^2^ is the Euclidean distance between interactive node *w*_*f*_ and clustering center *V*_*i*_.

Enhancing the performance of big data fusion and information clustering in interface interaction can be achieved through the big data processing method of interface interaction in the given study.

## 4. Experimental Results and Analysis

**Figure 3:**
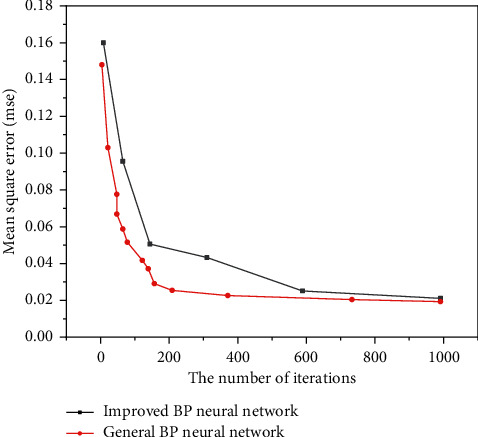
Comparison of the number of iterations of Berkeley data algorithms.

**Figure 4:**
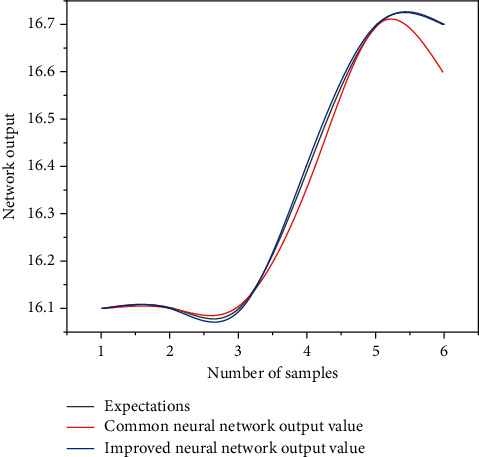
Comparison of Berkeley data test sample output.

In the experiment, the output vector of the data set has one dimension and the input vector has two dimensions. The data set contains 70 groups, including 50 training samples, 6 verification samples, and 6 test samples. The three-layer neural network is used, the sigmoid function is used as the excitation function in the hidden middle layer, and the excitation function in the output layer is selected as the linear function. The training comparison results of the two networks are shown in Figures 3 and 4. It can be seen from Figure 3 that in terms of convergence speed, the optimization algorithm given is faster than the average neural network.  Figure 4 shows the error value of the network after the improved BP neural network algorithm training. The error value is smaller than that of ordinary neural networks. In addition, after this algorithm is added to the evolution strategy, it can effectively accelerate the convergence speed and prevent it from falling into the local minimum [23].

In order to verify this method, the method proposed in this paper and other methods are used to deal with big data in the same financial industry. The time required for the two methods to process big data under different large data volumes is compared. The comparison results are shown in Table 1. The distributed sample set of big data is 200, the time interval of sample collection is 0.25 s, and the embedded distributed time delay of big data information processing is *τ* = 5.

**Figure 5:**
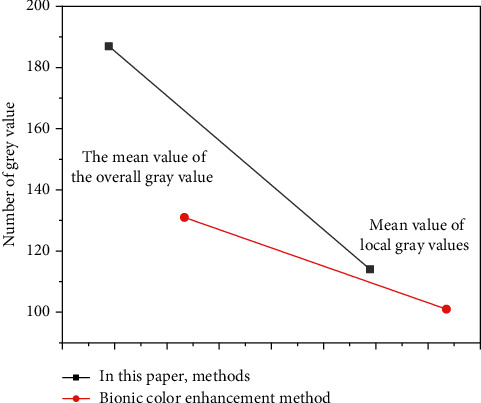
Comparison of mean gray value.

**Figure 6:**
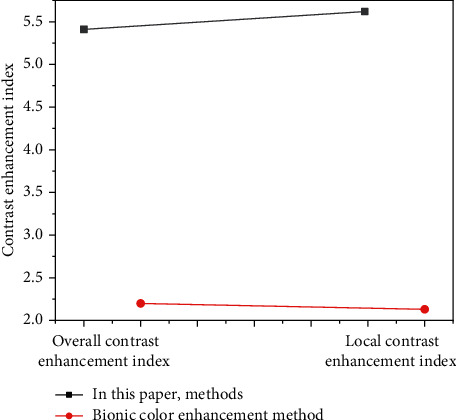
Contrast improvement index comparison.

As can be seen from Table 1 that with the increase of the amount of data, the processing time of the two methods of big data is increasing. When the amount of data is small, the processing time difference between the two methods is small. However, with the increase of the amount of data, the processing time of the methods used in this paper shows a steady growth trend, while the processing time of other methods increases faster. When the amount of data reaches 5000 GB, the method in this paper takes only 0.20 seconds, while other methods take 0.72 seconds. It can be seen that the big data processing efficiency of the method in this paper is relatively high. In order to measure the visual effect of the text and image of the web interface optimized and designed by the method in this paper, we compare the two methods to optimize the design of the average gray value of the text image of the web interface and the image contrast improvement index. The results are shown in Figures 5 and 6.

**Figure 7:**
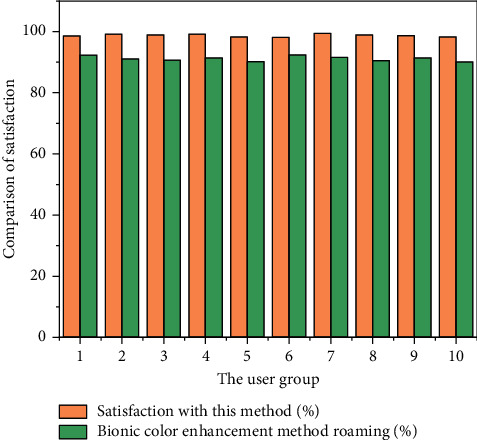
Comparison of user satisfaction.

As can be seen from Figure 5, the average value of the overall gray value and the average value of the local gray value of the web interface image optimized by this method are 186.34 and 111.34. The overall average gray value and the average local gray value of the image optimized by other methods are 131.56 and 96.42, respectively. The average gray value of the web interface image optimized by the method in this paper is higher than the average gray value of the image optimized by other methods. Therefore, the web interface image brightness by the optimized design of the method in this paper is better, showing better image visual effects. It can be seen from Figure 6 that the overall contrast improvement index and the partial improvement index of the web interface image optimized by the method in this article are 5.43 and 5.76. The overall contrast improvement index and the partial improvement index of the web interface image using other methods are 2.23 and 2.32, respectively. We prove that the visual effects of web interface images by the optimized design of the method in this paper are better than those by other methods, creating a more comfortable web interface image visual effect. In order to further verify the visual effect of web interface text after visual optimization, 200 users in the global resources network were randomly selected and divided into 10 groups on average. The satisfaction level of 10 groups of users with the visual effect of web interface text optimized by this method and other methods was measured. The comparison results are shown in Figure 7.

As can be seen from Figure 7, the user satisfaction of the visual effect of the web interface text optimized by this method is more than 98% and the user satisfaction of other methods is only about 90%. The visual satisfaction of the web interface text optimized by this method is significantly higher than that of the comparison method. The test results show that the web interface text visual optimization effect of this method is better and can meet the satisfaction needs of most users.

## 5. Conclusion

The visual optimization of web interface is performed to provide guarantee for better human-computer interaction. The visual optimization of images, words, and other texts of web interface can better provide visual experience for people and drive people's emotions. In order to transfer information between people and computers in the best and most effective way and better complete human-computer interaction, this paper adopts the method of color image enhancement and text optimization of the web interface based on visual characteristics to realize the optimization of text vision of the web interface. Through test and analysis, it can be seen that this method has good visual optimization effect.

## Figures and Tables

**Table 1:** Comparison of the time required to process big data.

Big data (GB)	The proposed method (s)	Other methods (s)
10	0.05	0.07
50	0.06	0.08
100	0.05	0.12
200	0.08	0.019
500	0.09	0.25
1000	0.12	0.28
2000	0.13	0.35
3000	0.15	0.36
4000	0.18	0.42
5000	0.20	0.72

## Data Availability

The data sets used to support the findings of this study are available from the author upon request.
